# A Study of Occurrence of Domestic Accidents in Semi-Urban Community

**DOI:** 10.4103/0970-0218.40878

**Published:** 2008-04

**Authors:** Dinesh J Bhanderi, Sushilkumar Choudhary

**Affiliations:** Department of Community Medicine, P.S. Medical College, Karamsad, Gujarat, India

**Keywords:** Domestic accidents, falls, semi-urban community

## Abstract

**Context:**

Domestic accidents are worldwide public health problems. The consequences of a domestic accident may prove disastrous as it may result in disability and loss of productivity. In this context, the present study was carried out to characterize the occurrence of domestic accidents in a semi-urban community.

**Aims:**

To study the incidence of domestic accident in a semi-urban community and its association with various epidemiological factors.

**Settings and Design:**

Community-based cross-sectional study of 796 households consisting of 4086 individuals residing in a semi-urban area.

**Materials and Methods:**

Complete information from 796 households consisting of 4086 individuals was collected through semi-structured, pre-tested questionnaire. Domestic accident was considered when any of these individuals had met with an accident inside the house or in the immediate surroundings of the house during the last 6 months from the date of survey. The collected data were tabulated and analyzed.

**Statistical Analysis Used:**

Simple proportions and Chi-squared test.

**Results:**

The incidence of domestic accidents was found to be 1.7%. The most common accident reported was fall. Occurrence of falls was found to be associated with age and overcrowding. Other accidents noted were burns, scalds, electrocution, injuries and accidental poisoning. Accidents were reported in significantly higher proportion in extreme age groups and in females. Higher proportion of accidents occurred during the morning and evening hours. About 10.1% were treated at home, 72.5% as outdoor patients and 17.4% as indoor patients. The mean duration of hospital stay was found to be 2 weeks. Full recovery was observed in 82.6% cases, whereas permanent disability was found in only 2.9% subjects, while 14.5% reported chronic pain after the accident. No death related to domestic accident was reported in the present study.

**Conclusions:**

Domestic accidents are more common in extreme age groups and in females. The reasons may be the higher amount of time spent at home and greater participation in daily home activities. Falls being the most frequent type of accidents, proper designing of house and adequate illumination may help in reducing their occurrence, as the majority of accidents occurred during the morning and evening hours in our study.

Domestic accident is an accident that takes place at home or in its immediate surroundings, and, more generally, all accidents not connected with traffic, vehicles or sport. Domestic accidents are worldwide public health problems. In some European countries, accidents at home kill more people than road accidents, in spite of strict safety regulations and laws regarding buildings and living areas.(1) The problem is more grave in developing countries, particularly in rural areas, shanty towns or informal dwellings.(2)

Every domestic accident brings a varying measure of distress to the victim as well as the family members. The consequences may be disastrous both for the individual and the society when the accident results in permanent disability, as the victim loses his earning capacity and may not be able to enjoy a normal active life. Children in particular are more vulnerable to domestic accidents, resulting in disability and loss of future productivity. In this context, the present study was carried out to characterize the occurrence of domestic accidents in a semi-urban community.

## Materials and Methods

The present descriptive study was conducted in two randomly selected wards of Petlad town in Anand district, Gujarat, from September 2005 to December 2005. There were total 832 households in these two wards. A household included all the persons who occupy a housing unit or a dwelling unit. Complete information of 796 households consisting of 4086 individuals was collected by interviewing the available eldest adult belonging to the particular household using a semi-structured, pre-tested questionnaire as the remaining 36 households could not be approached as their housing units were found locked during two consecutive visits. Domestic accident was considered when any of these individuals had met an accident inside the house or in the immediate surroundings of the house during the last 6 months from the date of survey. Overcrowding was considered when number of persons per room exceeded the accepted standards.(3) The collected data were tabulated and analyzed in terms of proportion using Epi 6 software. Chi-squared test was applied to study the relationship between occurrence of accidents and different socio-demographic variables. *P*-value less than 0.05 was considered significant.

## Results and Discussion

The total number of reported domestic accidents in our study was 69, making the incidence 1.7%. Devroey *et al.* reported an incidence of 2.7% in their study done in Belgium.(4) As shown in Table 1, the most common accident reported was fall, i.e., 71.0%. This category included fall on floor, slipping in bathroom, fall from height and fall from stairs. Other accidents noted were burns, scalds, electrocution, injuries and accidental poisoning. In accidental poisoning group, two cases of rat poison consumption and one case of cleansing acid consumption were reported. All the three cases were in the age group of 0-15 years. No case of drowning or suffocation was reported. Chaurasia and Shukul observed a higher proportion of burns and scalds in their study.(5) The LARES survey of the WHO Regional Office for Europe reported cuts as the most frequent accident type, followed by falls and burns(6); while burns and sharp-object injuries were the most common types of domestic accidents in the study by Neghab *et al*.(7) In our study, seven out of nine cases of burns and scalds were found in females. Table 2 depicts that occurrence of accidents was higher in the extreme age groups. i.e. in childhood and in old age. The association was statistically significant. Analyzing further the occurrence of accidents among the individuals in the age group of 0-15 years, it was found to be marginally higher though not significant, in the age group of 5-10 years [Table 3]. While categorizing occurrence of falls according to age group, it was observed in higher proportion in the age group of or more than 60 years, which was statistically significant [Table 4]. In our study, overcrowding was found in the case of 1615 individuals. Falls were reported in significantly higher number of individuals who were occupying overcrowded dwellings [Table 5]. Similarly, female gender was found to be a significant predictor of domestic accidents [Table 6]. Chaurasia and Shukul also reported a higher incidence of domestic accidents in females in their study except in the age group of more than 50 years.(5) Neghab *et al.* also reported similar gender difference in their study.(7) Though the proportion of accidents was found marginally higher in families with low monthly income, it was not statistically significant [Table 7], which was in contrast to the findings by Chaurasia and Shukul.(5)

**Table 1:** Occurrence of accidents according to type

Type of accident	Frequency no (%)
Falls	49 (71.0)
Burns	2 (2.9)
Scalds	7 (10.1)
Electrocution	1 (1.4)
Injury	7 (10.1)
Accidental poisoning	3 (4.3)
Total	69 (100.0)

**Table 2:** Occurrence of accidents according to age group

Age group (years)	Accident		Total no. (%)
		
	Yes no. (%)	No no. (%)	
0-15	27 (2.1)	1236 (97.9)	1263 (100.0)
15-30	9 (0.9)	1004 (99.1)	1013 (100.0)
30-45	12 (1.4)	837 (98.6)	849 (100.0)
45-60	10 (1.6)	629 (98.4)	639 (100.0)
> 60	11 (3.4)	311 (96.6)	322 (100.0)
Total	69 (1.7)	4017 (98.3)	4086 (100.0)

χ^2^ = 11.68, df = 4, *P* = 0.0199

**Table 3:** Occurrence of accidents in the age group of 0-15 years

Age group (years)	Accident		Total no. (%)
		
	Yes no. (%)	No no. (%)	
0-1	2 (1.6)	124 (98.4)	126 (100.0)
1-5	9 (2.2)	397 (97.8)	406 (100.0)
5-10	11 (2.8)	380 (97.2)	391 (100.0)
10-15	5 (1.5)	335 (98.5)	340 (100.0)
Total	27 (2.1)	1236 (97.9)	1263 (100.0)

χ^2^ = 1.77, df = 3, *P* = 0.6212

**Table 4:** Occurrence of falls according to age group

Age group (years)	Fall		Total no. (%)
		
	Yes no. (%)	No no. (%)
0-15	19 (1.5)	1244 (98.5)	1263 (100.0)
15-30	5 (0.5)	1008 (99.5)	1013 (100.0)
30-45	5 (0.6)	844 (99.4)	849 (100.0)
45-60	9 (1.4)	630 (98.6)	639 (100.0)
>60	11 (3.4)	311 (96.6)	322 (100.0)
Total	49 (1.2)	4037 (98.8)	4086 (100.0)

χ^2^ = 21.51, df = 4, *P* = 0.000

**Table 5:** Occurrence of falls according to presence of over crowding

Over crowding	Fall		Total no. (%)
		
	Yes no. (%)	No no. (%)
Present	31 (1.9)	1584 (98.1)	1615 (100.0)
Absent	18 (0.7)	2453 (18.3)	2471 (100.0)
Total	49 (1.2)	4037 (98.8)	4086 (100.0)

χ^2^ = 11.69, df = 1, *P* = 0.00063

**Table 6:** Gender-wise distribution of accidents

Gender	Accident		Total no. (%)
		
	Yes no. (%)	No no. (%)	
Male	23 (1.0)	2241 (99.0)	2264 (100.0)
Female	46 (2.5)	1776 (97.5)	1822 (100.0)
Total	69 (1.7)	4017 (98.3)	4086 (100.0)

χ^2^ = 13.84, df = 1, *P* = 0.00019

**Table 7:** Occurrence of accidents according to monthly income

Monthly income (Rupees)	Accident		Total no. (%)
		
	Yes no. (%)	No no. (%)	
<3000	26 (1.9)	1331 (98.1)	1357 (100.0)
3000-6000	23 (1.9)	1203 (98.1)	1226 (100.0)
6000-9000	14 (1.4)	1007 (98.6)	1021 (100.0)
>9000	6 (1.3)	476 (98.7)	482 (100.0)
Total	69 (1.7)	4017 (98.3)	4086 (100.0)

χ^2^ = 1.87, df = 3, *P* = 0.599

Considering the time of accident, 32 (46.4%) accidents occurred during th morning hours, 15 (21.7%) in the afternoon, 18 (26.1%) in the evening and 4 (7.8%) during the night. So, a higher proportion of accidents occurred during the morning and evening hours. As far as treatment-seeking pattern is concerned, out of 69 subjects, seven (10.1%) were treated at home, 50 (72.5%) as outdoor patients and 12 (17.4%) as indoor patients. The mean duration of hospital stay was found to be 2 weeks. Full recovery was observed in 57 (82.6%) cases of domestic accidents, while permanent disability was found in only two (2.9%) subjects in the form of short limb, while 10 (14.5%) reported chronic pain after the accident. Thus, in our study, minimal number of domestic accidents resulted in permanent disability. No death related to domestic accident was reported in the present study. Though Neghab *et al.* reported permanent disability rate of only 0.05%, mortality rate due to domestic accidents was quite high, i.e. 1.3% in their study.(7)

## Conclusion

Our study concludes that domestic accidents are more common in extreme age groups and in females. The reasons may be the higher amount of time spent at home and greater participation in daily home activities. Falls being the most frequent type of accidents, proper designing of house and adequate illumination may help in reducing their occurrence, as the majority of accidents occurred during the morning and evening hours in our study. Our study reported minimal disability resulting from domestic accidents, while no mortality was found. Such low rates may be the result of observations made in a semi-urban community where better health services are available and accessible. A broader study involving the rural population may provide a clearer picture of the epidemiology of domestic accidents in our country.
